# Association of the Dedicator of Cytokinesis 2 (DOCK2) Gene Polymorphisms with COVID-19 and Plasma LDH, AST, ALT, and Ferritin Levels

**DOI:** 10.3390/biom16050643

**Published:** 2026-04-25

**Authors:** José Manuel Fragoso, Rosalinda Posadas-Sánchez, Alberto López-Reyes, Laura E. Martínez-Gómez, Julian Ramírez-Bello, Giovanny Fuentevilla-Alvarez, Gilberto Vargas-Alarcón

**Affiliations:** 1 Departament of Molecular Biology, Instituto Nacional de Cardiología Ignacio Chávez, Mexico City 14080, Mexico; jose.fragoso@cardiologia.org.mx; 2 Department of Endocrinology, Instituto Nacional de Cardiología Ignacio Chávez, Mexico City 14080, Mexico; rosalinda.posadas@cardiologia.org.mx (R.P.-S.); miguel.fuentevilla@cardiologia.org (G.F.-A.); 3 Geroscience Laboratory, Instituto Nacional de Rehabilitación “Luis Guillermo Ibarra Ibarra”, Mexico City 14389, Mexico; alopez@inr.gob.mx (A.L.-R.); lemartinez@inr.gob.mx (L.E.M.-G.); 4 Clinical Research, Instituto Nacional de Cardiología Ignacio Chávez, Mexico City 14080, Mexico; julian.ramirez@cardiologia.org.mx; 5 Research Direction and Molecular Biology Department, Instituto Nacional de Cardiología Ignacio Chávez, Juan Badiano No. 1, Tlalpan, Mexico City 14080, Mexico

**Keywords:** COVID-19, genetics, dedicator of cytokinesis 2 (DOCK2), SARS-CoV-2, susceptibility

## Abstract

This case-control study investigated the association between polymorphisms in the dedicator of cytokinesis 2 (*DOCK2*) gene and susceptibility to COVID-19 in a Mexican population. Methods: Genotyping of five single-nucleotide polymorphisms (SNPs) in the *DOCK2* gene (rs9307 *A/G*, rs1045176 *G/T*, rs1045168 *C/T*, rs2112703 *A/C*, and rs2287727 *A/C*) was performed using TaqMan assays in 248 COVID-19 patients and 288 healthy controls. Results: No significant differences were observed in the allelic or genotypic distributions of rs1045176 *G/T* and rs2287727 *A/C* between cases and controls. However, under multiple genetic inheritance models (co-dominant, dominant, recessive, heterozygous, and additive), the rs9307 *A*, rs1045168 *C*, and rs2112703 *A* alleles were significantly associated with a reduced risk of COVID-19 (*p* < 0.05). Furthermore, sub-analyses stratified by genotype in COVID-19 patients revealed that the rs9307 *AA*, rs1045168 *CC*, and rs2112703 *AA* genotypes correlated with altered plasma concentrations of lactic acid dehydrogenase (LDH), alanine aminotransferase (ALT), aspartate aminotransferase (AST), and ferritin. Conclusions: The *DOCK2* SNPs rs9307 *A/G*, rs1045168 *C/T*, and rs2112703 *A/C* are associated with decreased susceptibility to COVID-19 in this population and influence plasma levels of LDH, ALT, AST, and ferritin, suggesting a potential role in disease pathogenesis and severity.

## 1. Introduction

Coronavirus disease 2019 (COVID-19) is caused by severe acute respiratory syndrome coronavirus 2 (SARS-CoV-2). As of 23 March 2024, a total of 777,684,506 confirmed cases and 7,092,720 deaths have been reported worldwide [1]. Genome-wide association studies (GWASs) and case-control studies have identified polymorphisms in various genes that encode molecules [2,3,4]—including transmembrane serine protease 2 (TMPRSS2), neutrophil-expressed protease (ELANE), angiotensin-converting enzyme 2 (ACE2), tolloid-like 1 (TLL1), ABO blood group antigens, cytokines, chemokines, major histocompatibility complex (MHC) classes I and II, tyrosine kinases, Toll-like receptors, and cathepsins B and L—that may play a significant role in SARS-CoV-2 infection susceptibility and severity [5,6,7,8,9]. However, most of these studies have focused primarily on Caucasian populations, underscoring the need for further research in non-European populations to better elucidate the genetic factors influencing COVID-19 outcomes.

Dedicator of cytokinesis 2 (DOCK2) is a guanine nucleotide exchange factor (GEF) that activates the small GTPase Rac1, facilitating GTP-GDP exchange [10]. DOCK2 plays a crucial role in immune regulation, particularly in lymphocyte activation and migration [11,12]. Mutations in the *DOCK2* gene have been linked to combined immunodeficiency, predisposing individuals to early-onset invasive bacterial and viral infections, including those caused by varicella-zoster virus, cytomegalovirus, mumps, adenovirus, and Pneumocystis jirovecii [13,14]. A recent large-scale GWAS in Japanese COVID-19 patients identified a *DOCK2* risk allele associated with severe disease in individuals under 65 years of age [15]. Furthermore, Namkoong et al. demonstrated, using an animal model, that DOCK2 suppression exacerbates COVID-19 severity and that its regulatory function modulates immune responses against SARS-CoV-2 infection [15]. Similarly, Dobbs et al. reported that autosomal recessive DOCK2 deficiency is associated with severe invasive pneumonia and combined immunodeficiency [13].

Given these findings, the objective of this study was to evaluate the association between *DOCK2* polymorphisms and COVID-19 susceptibility, as well as biochemical markers of SARS-CoV-2-related tissue damage, in the Mexican population.

## 2. Materials and Methods

### 2.1. Characteristics of the Study Population

This case-control study enrolled a total of 536 Mexican mestizo adults, comprising 248 RT-PCR-confirmed COVID-19 patients and 288 healthy controls, all of whom provided written informed consent. The patient cohort was prospectively recruited from three tertiary care centers (Instituto Nacional de Rehabilitación Luis Guillermo Ibarra Ibarra, Hospital Juárez de México, and Instituto Nacional de Cardiología Ignacio Chávez) between April 2020 and February 2021, capturing the initial pandemic wave in Mexico. All 248 COVID-19 patients were hospitalized at the time of enrollment.

COVID-19 diagnosis was established through both molecular confirmation (positive SARS-CoV-2 RT-PCR from nasopharyngeal swabs) and characteristic clinical presentation, including the pathognomonic symptoms of anosmia and ageusia, along with respiratory manifestations (dry cough, nasal congestion), systemic symptoms (fever, fatigue), and various other indicators of infection (diarrhea, chills, headache, musculoskeletal pain) [16,17].

The control group consisted of 288 healthcare professionals (59% male, 40.9% female; mean age 33.2 ± 7.6 years) actively working in COVID-19 intensive care units, including resident physicians, nursing staff, and laboratory personnel. To ensure appropriate negative controls, all participants in this group underwent rigorous screening using the Elecsys^®^ Anti-SARS-CoV-2 immunoassay (Roche Diagnostics, Roche, Rotkreuz, Switzerland), with seronegative results required for inclusion. This population represented an ideal comparison group due to their high exposure risk yet confirmed lack of infection. Although this group was selected to minimize misclassification and confounding, we recognize that it may represent a population with enriched resistance rather than a strictly general-population control.

### 2.2. Laboratory Analyses

All blood samples were processed in compliance with Mexican regulatory standards (NOR-007-SSA3-2011, NOM-087-SEMARNAT-SSA1-2002, NOM-010-SSA2-2010, NMX-EC-15189-IMNC-2015) using Class II biological safety cabinets under institutional biosafety protocols.

We assessed multi-organ damage through standardized biomarkers: The measurement of biochemical markers was performed at the time of patient admission.

- Renal/Hepatic Function: Creatinine (>1.3 mg/dL), ALT/AST (>35 U/L), total bilirubin (>1.2 mg/dL)
- Inflammatory/Hematologic Markers: Ferritin (>300 ng/dL), LDH (>160 U/L), C Reactive Protein (CRP) (>10 mg/dL), hemoglobin (>17 g/dL), platelets (>350 × 10^3^/μL)

Diagnostic thresholds followed the MSD Manual Professional Version 2018 [https://www.msdmanuals.com/professional/SearchResults?query=Normal+laboratory+values (accessed on 2 January 2025)].

Comorbidity definitions included:

- Type 2 Diabetes mellitus (T2DM): Fasting glucose ≥ 126 mg/dL, physician diagnosis, or active antidiabetic treatment [https://www.msdmanuals.com/professional/endocrine-and-metabolic-disorders/diabetes-mellitus-and-disorders-of-carbohydrate-metabolism/diabetes-mellitus-dm#v29299021 (accessed on 2 January 2025)];
- Hypertension: Systolic Blood Pressure (SBP) ≥ 130 mmHg, diastolic Blood Pressure (DBP) ≥ 80 mmHg, or antihypertensive medication use.

### 2.3. Genetic Analysis

Genomic DNA was isolated from peripheral blood samples using standardized methods [18]. We genotyped five *DOCK2* SNPs (rs9307 *A/G*, rs1045176 *G/T*, rs1045168 *C/T*, rs2112703 *A/C*, and rs2287727 *A/C*) in both COVID-19 patients and controls using TaqMan allelic discrimination assays on a QuantStudio 12K Flex Real-Time PCR system (Applied Biosystems, Foster City, CA, USA). Complete SNP characteristics, including chromosomal positions (GRCh38), nucleotide substitutions, minor allele frequencies, and genomic position, are provided in Supplementary Table S1.

### 2.4. In Silico Functional Characterization of SNPs

The regulatory potential of selected SNPs was investigated through comprehensive in silico analyses utilizing established bioinformatics platforms (SNPinfo [19], RegulomeDB v2.2 [20], and HaploReg v4.2 [21]). These computational tools enabled systematic evaluation of putative functional consequences, including alterations to transcription factor binding motifs and modifications to histone marks. Evolutionary conservation patterns were quantitatively assessed using SiPhy algorithms, with positive conservation scores identifying phylogenetically constrained genomic regions.

Subsequent functional characterization employed protein-protein interaction (PPI) network analysis via STRING v12.0 [22]. Network construction incorporated genes associated with SNP-modified transcription factor binding sites, applying a stringent interaction confidence threshold (score ≥ 0.7) to integrate multiple evidence streams: experimentally validated interactions, curated database annotations, co-expression patterns, and literature-derived associations.

Gene Ontology (GO) enrichment analysis was performed within the STRING environment to elucidate affected biological pathways. The analysis systematically evaluated three ontological domains: Biological Processes (BPs), Molecular Functions (MFs), and Cellular Components (CCs). Statistically significant functional terms (FDR-adjusted *p*-value < 0.05) were identified and organized into functional clusters, with particular emphasis on immune response pathways, inflammatory signaling cascades, and metabolic regulation networks.

### 2.5. Statistical Analysis

The Hardy-Weinberg equilibrium was assessed using chi-square tests. Data distribution normality was evaluated with the Shapiro-Francia test. Continuous variables were compared between COVID-19 patients and controls using Student’s *t*-test (presented as mean ± SD) for normally distributed data or the Mann-Whitney U test (presented as median [interquartile range]) for non-normally distributed data. Categorical variables were analyzed using Fisher’s exact test or chi-square tests as appropriate. Associations between *DOCK2* SNPs and COVID-19 susceptibility were evaluated under five genetic models: additive, codominant, dominant, heterozygous, and recessive [23,24]. These analyses were performed using logistic regression adjusted for age and gender as confounding variables. *p*-values were corrected for multiple testing using the Bonferroni method based on the number of tests performed for each SNP across inheritance models. Results are presented as odds ratios (ORs) with 95% confidence intervals. The study power was set at 0.80 (calculated using OpenEpi; http://www.openepi.com/SampleSize/SSCC.htm, (accessed on 15 January 2026)), with statistical significance defined as *p* < 0.05.

To examine potential associations between *DOCK2* genotypes and biochemical markers of SARS-CoV-2-induced tissue damage, COVID-19 patients were stratified by genotype. Between-group comparisons were performed using ANOVA after logarithmic transformation of non-normally distributed variables. Variance homogeneity was verified using Levene’s test and confirmed by the F-test.

Haplotype analysis and linkage disequilibrium (LD) estimation were performed using Haploview version 4.1 (Broad Institute of MIT and Harvard, Cambridge, MA, USA). This software utilizes the HapMap project database and the expectation-maximization (EM) algorithm to reconstruct haplotypes by determining the most likely combination of alleles that co-segregate due to physical proximity. The analysis generated maximum-likelihood estimates of LD measures, including D′, logarithm of odds (LOD) scores, and r^2^ [25].

### 2.6. Ethical Statement

The study strictly adhered to the ethical principles of the Declaration of Helsinki and received formal approval from the Ethics, Biosecurity, and Research Committees of the Instituto Nacional de Cardiología Ignacio Chávez (Project No. 20-1202, approved 8 January 2021), Hospital Juárez de México (Project No. HJM 024/22-I, approved 6 October 2022), and Instituto Nacional de Rehabilitación Luis Guillermo Ibarra Ibarra (Project No. 17/20 AC, approved 1 June 2020). All study participants provided written informed consent in full compliance with institutional ethical guidelines.

## 3. Results

### 3.1. Characteristics of the Study Sample

Table 1 presents the demographic profile, clinical symptoms, comorbidities, and biochemical markers of the COVID-19 patients. The data demonstrate significantly elevated levels of acute-phase reactants, including ferritin, LDH, and CRP, along with increased ALT and AST enzymes. The most frequent clinical manifestations included cough (68.9%), dyspnea (61.6%), headache (25%), myalgia (26.2%), and fatigue (26.2%), with characteristic fever presentation (mean 37 ± 1.1 °C) and reduced oxygen saturation (87 ± 12%). These clinical and laboratory findings align with established COVID-19 diagnostic criteria as outlined in the MSD Manual Professional Version [https://www.msdmanuals.com/professional/SearchResults?query=Normal+laboratory+values (accessed on 2 January 2025)].

### 3.2. Association of DOCK2 Polymorphisms with COVID-19

The distribution of *DOCK*2 genotypes in both study groups (COVID-19 patients and controls) was in Hardy-Weinberg equilibrium (*p* > 0.05). With the exception of rs1045176 and rs2287727 SNPs, significant differences in allele and genotype frequencies were observed for rs9307 *A/G*, rs1045168 *C/T*, and rs2112703 *A/C* polymorphisms when comparing COVID-19 patients with controls (*p* < 0.05; Supplementary Table S2). Analysis of these COVID-19-associated SNPs under various inheritance models (Table 2) revealed: for rs9307 *A/G*, individuals carrying one or two copies of the *A* allele demonstrated reduced risk of COVID-19 under codominant 2 (OR = 0.37, *p* = 0.015), dominant (OR = 0.67, *p* = 0.023), recessive (OR = 0.42, *p* = 0.018), and additive (OR = 0.67, *p* = 0.005) models; for rs1045168 *C/T*, carriers of one or two *C* allele copies showed protective effects under codominant 1 (OR = 0.58, *p* = 0.005), dominant (OR = 0.55, *p* = 0.002), heterozygous (OR = 0.61, *p* = 0.011), and additive (OR = 0.59, *p* = 0.001) models; and for rs2112703 *A/C*, possession of one or two *A* allele copies was associated with decreased COVID-19 susceptibility under codominant 1 (OR = 0.54, *p* = 0.018), dominant (OR = 0.52, *p* = 0.012), heterozygous (OR = 0.55, *p* = 0.022) and additive (OR = 0.51, *p* = 0.007) models.

### 3.3. Haplotype Analysis

The haplotype analysis is shown in Table 3. This analysis was carried out according to chromosomal position. Based on this analysis, the rs2112703 *A/C*, rs2287727 *A/C*, rs1045168 *C/T*, rs1045176 *G/T*, and rs9307 *A/G* SNPs of the *DOCK2* gene were not in linkage disequilibrium (D′ < 0.40). Nonetheless, the results showed that the “*CATTG*” haplotype was more frequent in COVID-19 patients (31.6%) than in controls (21.7%), suggesting that this haplotype may increase the risk of developing COVID-19 (OR = 1.66, *p* < 0.001). In contrast, the “*AATTA*” haplotype was more frequent in controls (2.4%) than in COVID-19 patients (0.9%), suggesting that this haplotype may decrease the risk of COVID-19 (OR = 0.32, *p* = 0.019) (Table 3).

### 3.4. Association of DOCK2 Polymorphisms with Biochemical Markers of Damage

Data in the literature have shown that biochemical markers such as creatinine, ferritin, LDH, CRP, total bilirubin, ALT, AST, hemoglobin, and platelets may be altered by SARS-CoV-2 infection [19,20]. In this context: carriers of the rs9307 *AA* genotype had high ferritin levels (*p* < 0.05) and low LDH levels (*p* < 0.05) (Table 4); the rs1045168 *CC* genotype was associated with higher concentrations of ferritin, LDH, and total bilirubin (*p* < 0.05) (Table 4); and the rs2112703 *CA* genotype was associated with decreased ALT and AST levels (*p* < 0.05) (Table 4).

### 3.5. Bioinformatics Analysis for Functional Prediction

In silico analysis identified regulatory motif alterations in two of the three significant SNPs. The rs9307 variant, located in the 3′UTR region of *DOCK2*, was found to disrupt a binding motif for SETDB1, a key epigenetic regulator involved in chromatin remodeling processes. Similarly, the synonymous variant rs2112703 demonstrated the capacity to alter binding motifs for three critical transcription factors: CTCF (essential for chromatin organization), NRSF (a neuronal gene regulator), and Pax-5 (crucial for B-cell development), as detailed in Supplementary Table S3.

**Figure 1:**
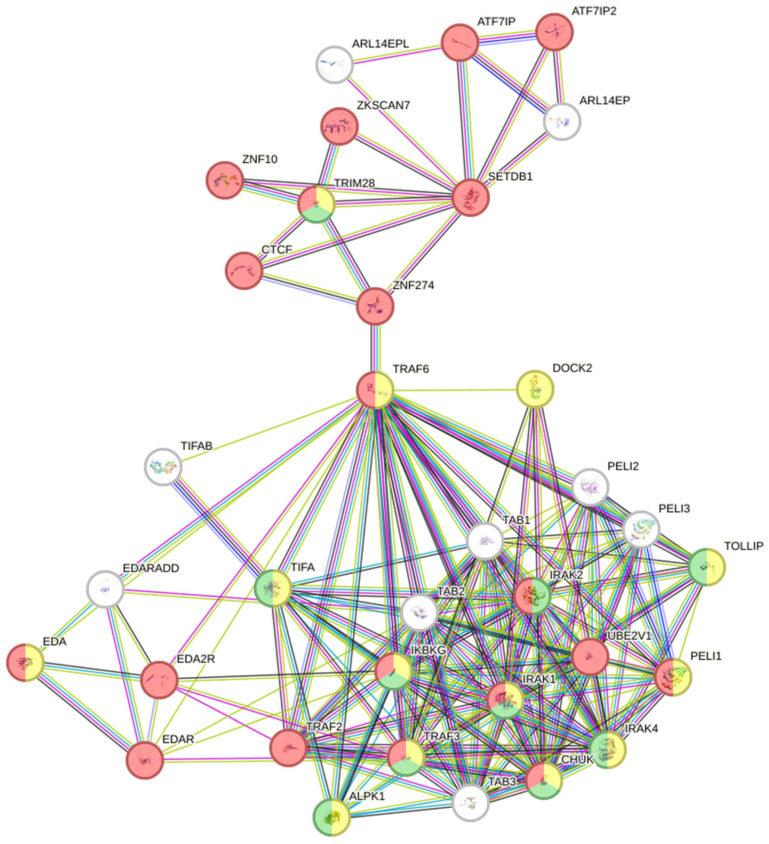
Protein-protein interaction network and functional enrichment analysis of DOCK2 and associated genes. The STRING-generated network illustrates interactions between DOCK2 and immune-related or transcriptional regulators. Colored edges represent different types of interaction evidence, including co-expression, physical interactions, and computational predictions. Node colors indicate functional relevance: red denotes transcriptional regulators, yellow indicates immune system process, green represents defense response. The analysis underscores the involvement of DOCK2 in immune signaling, inflammation, and metabolic regulation, suggesting its potential impact on disease outcomes.

Figure 1 presents the protein-protein interaction (PPI) network constructed using STRING, revealing significant interactions among genes associated with the identified SNPs (DOCK2, SETDB1, CTCF, NRSF, and Pax-5). In this network, protein nodes are connected by edges representing functional or physical interactions, with color-coding indicating biological functions: red nodes denote transcription factors involved in gene expression control, yellow marks immune system-related genes, and green identifies genes associated with defense responses. The clustering pattern reveals strong functional relationships between DOCK2 and immune signaling pathways, with additional regulatory influence from SETDB1 through its chromatin accessibility modifications.

Functional enrichment analysis of the PPI network genes (Supplementary Figure S1) showed significant representation in biological processes related to immune activation, inflammatory responses, and transcriptional regulation. Notably enriched pathways included immune system processes and cytokine signaling, both strongly associated with DOCK2’s established role in immune cell migration and activation. The analysis also highlighted epigenetic regulation and chromatin remodeling pathways linked to SETDB1’s histone methylation activity, along with transcriptional regulation of immune-related genes corresponding to CTCF, NRSF, and Pax-5 functions.

## 4. Discussion

Under multiple genetic inheritance models (co-dominant, dominant, recessive, heterozygous, and additive), the rs9307 *A*, rs1045168 *C*, and rs2112703 *A* alleles were significantly associated with a reduced risk of COVID-19. When COVID-19 patients were grouped by genotype, our data demonstrated that the rs9307 *AA*, rs1045168 *CC*, and rs2112703 *CA* genotypes significantly influence plasma levels of LDH, ALT, AST, and ferritin. While no prior experimental studies have examined the effects of these specific SNPs on these biochemical markers [26,27] our findings align with established evidence that SARS-CoV-2 infection alters multiple biochemical parameters.

The rs9307 variant is located in the 3′UTR region of *DOCK2*, which is crucial for microRNA (miRNA) binding, influencing mRNA stability and translation efficiency. SNPs in this region can modify miRNA binding sites, leading to either increased degradation or enhanced transcript stability [28,29]. Specifically, the rs9307 *AA* genotype may interfere with miRNA binding, potentially altering *DOCK2* expression patterns. As a guanine nucleotide exchange factor essential for immune cell activation and cytoskeletal regulation [30], altered *DOCK2* expression could significantly impact COVID-19 progression by either exacerbating hyperinflammation (through overexpression) [31] or impairing viral clearance (through underexpression) [32].

Furthermore, the results show that rs9307 is associated with the alteration of a binding motif for SETDB1, an epigenetic regulator that modulates chromatin states and gene expression [33]. Disruption of SETDB1 binding could affect the transcriptional repression of immune-related genes [34], further influencing *DOCK2* expression and immune response dynamics. Although rs9307 does not localize within a CpG island or directly alter a CpG dinucleotide, its regulatory impact appears to be mediated through other epigenetic mechanisms. Additionally, the observed association between the rs9307 *AA* genotype and increased ferritin levels suggests a link between altered *DOCK2* expression and systemic inflammation. Ferritin is an acute-phase reactant elevated in severe COVID-19 cases, reflecting heightened inflammatory activity [35]. In contrast, the same genotype was associated with lower LDH levels, a marker of cellular damage and hypoxia. This divergent pattern of high ferritin with low LDH suggests that rs9307 may modulate the balance between inflammation and tissue injury, contributing to the observed protective effect against severe COVID-19 [36]. These findings align with previous reports highlighting the role of DOCK2 in immune regulation and viral response [37]. Altered DOCK2 signaling, potentially due to changes in miRNA binding and SETDB1 regulatory function, may contribute to a more severe immune response in COVID-19.

The rs1045168 *C/T* SNP, although not located in a regulatory region, was also associated with biochemical markers. The rs1045168 *CC* genotype was associated with significantly elevated ferritin, LDH, and total bilirubin levels. In COVID-19, the concurrent elevation of these three markers is clinically relevant: ferritin reflects systemic inflammation and macrophage activation, LDH indicates cellular damage and hypoxia, and bilirubin points to hepatic involvement or hemolysis. The clustering of these alterations in *CC* carriers suggests a coordinated inflammatory and multi-organ stress response [38]. While rs1045168 is a synonymous variant, recent studies indicate that such a mutation can affect mRNA stability, splicing, or translation efficiency, potentially altering protein expression [39]. This SNP may influence DOCK2 protein folding or translation speed, subtly modulating its activity. The positive SiPhy score for rs1045168 suggests that this site is highly conserved, reinforcing its potential functional relevance [40]. The association of the *CC* genotype with increased markers of cellular damage and inflammation suggests a possible impact on disease progression by modifying the immune response. Similar associations between *DOCK2* variants and inflammatory markers have been reported in other infectious and inflammatory diseases, further supporting our findings. Such alterations may contribute to a stronger or prolonged inflammatory response, exacerbating the cytokine storm syndrome that worsens COVID-19 prognosis [41].

The rs2112703 *A/C* SNP was also significantly associated with biochemical markers in COVID-19 patients. Our data showed that the rs2112703 *CA* genotype was associated with decreased ALT and AST levels, indicating a potential attenuation of liver damage. Elevated transaminases are common in COVID-19 and are associated with hepatic injury, as a direct effect of either viral infection, systemic inflammation, or drug-induced hepatotoxicity [42]. Notably, the *CA* genotype, which carries the protective *A* allele, was associated with lower transaminase levels, suggesting that this variant may confer a protective effect against COVID-19-related hepatic dysfunction [43].

Importantly, rs2112703 is associated with alterations in binding motifs for CTCF, NRSF, and Pax-5. CTCF is a key insulator protein involved in chromatin organization and gene regulation [44], while NRSF regulates neuronal gene expression and may play a role in immune signaling [45]. Pax-5 is crucial for B cell development and immune function [46]. The positive SiPhy score for rs2112703 indicates high conservation, supporting its functional importance. Disruption of these motifs suggests that rs2112703 could modulate immune and inflammatory responses at the transcriptional level, potentially affecting *DOCK2* expression and function.

These findings align with the STRING network analysis, highlighting key interactions between DOCK2, immune-related genes, and transcriptional regulators. Additionally, gene ontology enrichment analysis reinforces the involvement of DOCK2 in immune system processes, cellular stress responses, and defense mechanisms. The presence of SNPs in *DOCK2* may disrupt these pathways, contributing to the variability in COVID-19 susceptibility and severity, supporting further investigation of *DOCK2* as a potential genetic marker for disease outcomes. Collectively, the associations between *DOCK2* SNPs and plasma levels of ferritin, LDH, bilirubin, ALT, and AST support a model in which genetic variation in this immune-related gene influences the host inflammatory response to SARS-CoV-2 infection. Although causal relationships cannot be established from association data alone, the consistency of these genotype-biomarker correlations across multiple markers—reflecting different aspects of COVID-19 pathophysiology (inflammation, tissue damage, hepatic involvement)—strengthens the hypothesis that *DOCK2* variants contribute to disease susceptibility and severity through modulation of immune activation and inflammatory cascades. Although our in-silico analyses and genotype-biomarker associations provide mechanistic insights, experimental functional studies are required to definitively establish the causal links between these *DOCK2* SNPs and the observed immune response phenotypes.

Beyond these functional considerations, it is also important to note that allele frequencies differed significantly between Mexican mestizos and other populations (Supplementary Table S4), particularly for: rs2112703 *A*, rs2287727 *A*, rs1045168 *C* (lower in Mexicans vs. Caucasians), rs1045176 *G* (lower in Mexicans and Caucasians vs. Asians/Africans) and rs9307 *A* (similar across Mexicans, Caucasians, and Asians). These ethnic variations highlight the need for multicenter studies to clarify population-specific genetic effects.

This study has some limitations. Although we identified significant associations between *DOCK2* SNPs and biochemical markers and performed in silico analyses suggesting potential regulatory effects, we did not conduct experimental functional assays to validate these predicted mechanisms. Additionally, we did not quantify circulating cytokine levels, which would have provided a more direct link between genotypes and the inflammatory response. Although the statistical power for the main analyses was set at 0.80, the sample size warrants replication in larger cohorts to confirm these findings. Finally, the models were adjusted only for age and gender.

## 5. Conclusions

In conclusion, our study identifies: protective associations of rs9307 *A/G*, rs1045168 *C/T*, and rs2112703 *A/C* (individually and as the *AATTA* haplotype) to COVID-19; significant genotype-biomarker correlations (rs9307 *AA*, rs1045168 *CC,* and rs21112703 *AC* genotypes with LDH, transaminases, and ferritin levels); and Mexican population-specific allele distributions warranting further investigation in other ethnic groups.

## Figures and Tables

**Table 1:** Demographic, biochemical, and clinical parameters of the COVID-19 patients.

Characteristics	COVID-19 Patients (n = 248)
Age (years)	56.34 ± 13.80
Sex n (%)	172 (69.3) Male
	76 (30.6) Female
Clinical symptoms	
Cough n (%)	171 (68.9)
Dyspnea n (%)	153 (61.6)
Chest Pain n (%)	24 (9.6)
Headache n (%)	62 (25)
Myalgia n (%)	65 (26.2)
Fatigue n (%)	65 (26.2)
Abdominal Pain n (%)	11 (4.4)
Nausea n (%)	13 (5.2)
Emesis n (%)	14 (5.6)
Diarrhea n (%)	30 (12.1)
Odynophagia n (%)	45 (18.1)
Fever n (%)	94 (37.9)
Rhinorrhea n (%)	10 (4.0)
Invasive mechanical ventilation	70 (28.2)
Temperature °C	37 ± 1.1
Oxygen saturation (SpO_2_)	87 ± 12
Heart rate	87 ± 20
Comorbidities	
Obesity n (%)	187 (75.4)
TDM2 n (%)	89 (35.8)
Hypertension n (%)	106 (42.7)
Biochemical markers	
Creatinine (mg/dL)	0.89 [0.67–1.31]
Total bilirubin (mg/dL)	0.60 [0.46–0.89]
Ferritin (ng/μL)	653 [356–1086]
LDH (U/dL)	332 [239–452]
Protein C Reactive (mg/dL)	27.0 [7.3–131]
ALT (U/dL)	40.2 [24.1–69.3]
AST (U/dL)	40 [26–62]
Hemoglobin (g/dL)	13.8 [11.3–15.2]
Platelets (10^9^/L)	275 [200–351]

Abbreviations: LDH, Lactic acid dehydrogenase; ALT, Alanine transaminase; AST, aspartate aminotransferase. Data are expressed as mean ± standard deviation, percentage, and median-percentiles (25th–75th).

**Table 2:** Association of *DOCK2* SNPs with COVID-19.

	n (Genotype Frequency)			MAF	Model	OR (95%CI)	*pC*
*DOCK2*	rs9307 *A/G*						
Control	*GG*	*GA*	*AA*	*A*	Co-dominant 1	0.77 (0.53–1.09)	0.148
(n = 288)	144 (0.500)	118 (0.409)	26 (0.090)	0.295	Co-dominant 2	0.37 (0.17–0.80)	**0.015**
					Dominant	0.67 (0.47–0.95)	**0.023**
					Recessive	0.42 (0.20–0.89)	**0.018**
COVID-19	146 (0.589)	92 (0.371)	10 (0.040)	0.225	Heterozygous	0.82 (0.57–1.16)	0.259
(n = 248)					Additive	0.67 (0.51–0.89)	**0.005**
*DOCK*2	rs1045168 *C/T*						
Control	*TT*	*CT*	*CC*	*C*	Co-dominant 1	0.58 (0.40–0.85)	**0.005**
(n = 288)	177 (0.615)	98 (0.340)	13 (0.045)	0.215	Co-dominant 2	0.35 (0.12–1.04)	0.056
					Dominant	0.55 (0.38–0.80)	**0.002**
					Recessive	0.41 (0.15–1.19)	0.085
COVID-19	183 (0.738)	60 (0.242)	5 (0.020)	0.141	Heterozygous	0.61 (0.42–0.89)	**0.011**
(n = 248)					Additive	0.59 (0.42–0.81)	**0.001**
*DOCK2*	rs2112703 *A/C*						
Control	*CC*	*AC*	*AA*	*A*			
(n = 288)	238 (0.826)	48 (0.167)	2 (0.070)	0.090	Co-dominant 1	0.54 (0.32–0.92)	**0.018**
					Dominant	0.52 (0.31–0.87)	**0.012**
					Heterozygous	0.55 (0.32–0.93)	**0.022**
COVID-19	224 (0.903)	24 (0.097)	0 (0.0)	0.048	Additive	0.51 (0.31–0.85)	**0.007**
(n = 248)							

Abbreviations: COVID-19, severe acute respiratory syndrome coronavirus 2; MAF, Minor allele frequency; OR, odds ratio; CI, confidence interval; *pC*, p-corrected by Bonferroni. The *p*-values were calculated by the logistic regression analysis adjusted by age and gender. Significant *p* values are in bold.

**Table 3:** Distribution of *DOCK2* haplotypes in COVID-19 patients and healthy controls.

Polymorphic Site (rsID-Number)	COVID-19 Patients n = 247	Healthy Controls n = 288	OR	95%CI	*p*
H: rs211270-rs2287727-rs1045168-rs1045176-rs9307	Hf	Hf			
H1 *(C A T T G)*	0.316	0.217	1.66	1.26–2.19	**<0.001**
H2 *(C C T T G)*	0.165	0.134	1.29	0.92–1.80	0.069
H3 *(C A T T A)*	0.118	0.125	0.92	0.64–1.34	0.347
H4 *(C A T G G)*	0.102	0.089	1.15	0.76–1.74	0.239
H5 *(C C T T A)*	0.074	0.098	0.72	0.47–1.13	0.078
H6 *(C C T G G)*	0.054	0.064	0.84	0.50–1.40	0.255
H7 *(C A C T G)*	0.050	0.068	0.73	0.43–1.23	0.120
H8 *(C A C G G)*	0.025	0.041	0.59	0.29–1.21	0.075
H9 *(C A C T A)*	0.019	0.037	0.54	0.25–1.17	0.057
H10 *(C C C T G)*	0.026	0.031	0.83	0.40–1.72	0.315
H11 *(A A C T G)*	0.017	0.026	0.61	0.25–1.46	0.134
H12 *(A A T T A)*	0.009	0.024	0.32	0.10–1.00	**0.019**

Abbreviations: Hf, Haplotype frequency; *p* = *p*-value. The polymorphism order of haplotypes is according to the position in the chromosome. (rs2112703-rs2287727-rs1045168-rs1045176-rs9307 chromosome 5q35). Significant *p* values are in bold.

**Table 4:** Association of the *DOCK2* gene SNPs with damage biochemical markers in the COVID-19 patients.

	SNP/Genotypes			
*DOCK2*	*rs9307 A/G*			
	*GG* (n = 146)	*GA* (n = 92)	*AA* (n = 10)	*p*
Creatinine (mg/dL)	0.86 [0.67–1.37]	0.89 [0.62–1.20]	1.09 [0.96–2.28]	0.540
Ferritin (ng/μL)	658 [377–1121]	615 [346–1039]	1042 [675–5972]	**0.026**
LDH (U/dL)	343 [256–454]	308 [232–408]	295 [271–433]	**0.040**
C-reactive protein (mg/dL)	27.9 [6.2–126]	27.8 [10–143]	19 [5.6–95]	0.684
Bilirubin Total (mg/dL)	0.60 [0.45–0.88]	0.60 [0.49–0.92]	0.71 [0.45–0.79]	0.818
ALT (U/dL)	41 [24–68]	38 [24–70]	30.2 [21–81]	0.775
AST (U/dL)	38.4 [26.5–62]	41.3 [25–62]	38 [22–57]	0.750
Hemoglobin (g/dL)	13.9 [11.6–15.3]	13.6 [11–15]	14.1 [11–12.3]	0.439
Platelets (10^−6^/μL)	279 [210–357]	266 [188–340]	235 [188–295]	0.906
Heart rate	85.7 ± 17.9	88.7 ± 20.5	92.7 ± 37.2	0.352
Temperature °C	36.7 ± 0.87	36.6 ± 1.42	36.8 ± 1.10	0.739
Oxygen saturation (SpO_2_)	85.7 ± 12.6	88.3 ± 10.5	89.6 ± 3.66	0.183
*DOCK2*	rs1045168 *C/T*			
	*TT* (n = 183)	*TC* (n = 60)	*CC* (n = 5)	*p*
Creatinine (mg/dL)	0.87 [0.68–1.27]	0.92 [0.63–1.42]	0.90 [0.87–1.03]	0.476
Ferritin (ng/μL)	636 [328–1069]	646 [380–1069]	2925 [1331–4391]	**0.007**
LDH (U/dL)	331 [239–457]	331 [258–411]	686 [549–819]	**0.012**
C-reactive protein (mg/dL)	25.4 [7.6–124]	40.5 [7.1–146]	59.6 [26–88]	0.670
Total bilirubin (mg/dL)	0.60 [0.44–0.82]	0.69 [0.57–1.14]	0.70 [0.53–0.77]	**0.004**
ALT (U/dL)	39 [23–71]	44 [27–64]	54.4 [41–61]	0.763
AST (U/dL)	38.4 [25–61]	40 [26–63]	80 [43–81]	0.343
Hemoglobin (g/dL)	13.7 [11–15]	13.9 [11.4–15.4]	16.4 [12.4–16.8]	0.196
Platelets (10^−6^/μL)	276 [206–355]	257 [195–346]	238 [209–293]	0.670
Heart rate	87.4 ± 19.4	87 ± 19.9	75.8 ± 23.2	0.429
Temperature °C	36.7 ± 1.18	36.7 ± 0.91	36.4 ± 0.42	0.836
Oxygen saturation (SpO_2_)	87.2 ± 11.2	86 ± 12.4	84.6 ± 17.9	0.740
*DOCK2*	rs2112703 *A/C*			
	*CC* (n = 224)	*CA* (n = 24)		*p*
Ferritin (ng/μL)	615 [357–1185]	786 [358–1070]		0.492
LDH (U/dL)	332 [241–453]	333 [237–374]		0.576
C-reactive protein (mg/dL)	29.5 [7.2–129]	21 [11.6–225]		0.831
Total bilirubin (mg/dL)	0.60 [0.44–0.88]	0.68 [0.50–0.93]		0.465
ALT (U/dL)	41 [24–71]	30 [17–50]		**0.021**
AST (U/dL)	47.5 [28–81]	33 [23–48]		**0.037**
Hemoglobin (g/dL)	13.6 [11.6–15.1]	14.3 [12.6–15.3]		0.418
Platelets (10^−6^/μL)	276 [200–355]	258 [189–330]		0.392
Heart rate	87.2 ± 19.9	85.7 ± 16.4		0.704
Temperature °C	36.7 ± 1.13	36.5 ± 0.93		0.403
Oxygen saturation (SpO_2_)	86.7 ± 12.6	88 ± 9.1		0.632

Abbreviations: LDH = lactic acid dehydrogenase, ALT = aminotransferase alanine, AST = aminotransferase aspartate. Data on heart rate, temperature, and oxygen saturation are expressed as mean ± standard deviation. The creatinine, ferritin, LDH, CRP, total bilirubin, ALT, AST, hemoglobin, and platelets are expressed in median-percentiles (25–75th). Significant *p* values are in bold. ANOVA and F-test were used.

## Data Availability

Due to confidentiality agreements, the data underlying this study are not publicly available. Access to the data can be requested through gilberto.vargas@cardiologia.org.mx, following their confidentiality protocols.

## Supplementary Materials

The following supporting information can be downloaded at https://www.mdpi.com/article/10.3390/biom16050643/s1, Supplementary Table S1. Information on the studied polymorphism tested; Supplementary Table S2. Allele and genotype frequencies of *DOCK2* gene polymorphisms in COVID-19 patients and healthy controls; Supplementary Table S3. In Silico Functional Prediction for Variants *DOCK2*; Supplementary Table S4. Allele frequencies (af) of the *DOCK2* SNPs in different populations; Supplementary Figure S1. Biological Process enrichment.
