# Supplementary material for: Association of the Dedicator of Cytokinesis 2 (DOCK2) Gene Polymorphisms with COVID-19 and Plasma LDH, AST, ALT, and Ferritin Levels
Source: Biomolecules. 2026 Apr 25;16(5):643. doi: 10.3390/biom16050643 (PMC13204226; doi:10.3390/biom16050643)
Supplement: Supplementary file 1 [file biomolecules-16-00643-s001.zip › biomolecules-4213724-supplementary.pdf]

Supplementary Table S1. Information of the studied polymorphism tested

**Gene symbol**	**SNP (rsID-number) [a,b]**	**Chromosome**	**Chromosome position**	**MAF**	**Location in gene**
DOCK2	rs2112703	5p14	169700093	*A*	Synonymous Variant Thr 404 Thr
DOCK2	rs2287727	5p14	170008709	*C*	Synonymous Variant Leu 1065 Leu
DOCK2	rs1045168	5p14	170077739	*C*	Synonymous Variant Arg 1632 Arg
DOCK2	rs1045176	5p14	170083016	*G*	3'UTR variant c*158
DOCK2	rs9307	5p14	170083019	*A*	3'UTR variant c*161

[a] Single Nucleotide Polymorphisms (rsID-number) in database dbSNP; [b] Given name according to NCBI, Current Build 156 (released September 21, 2022) https://www-ncbi-nlm-nih-gov.pbidi.unam.mx:2443/snp/rs1045176#variant_details.
MAF: Minor allele frequency.

Supplementary Table S2. Allele and genotype frequencies of *DOCK2* gene polymorphisms in COVID-19 patients and healthy controls

Polymorphic Site (rsID-number)	COVID-19 n=248 (n[%])	Controls n=288 (n[%])	*p
rs9307 *A/G*			
Allele			
G	384 (77.4)	406 (70.4)	
A	112 (22.5)	170 (29.5)	0.010
Genotype			
GG	146 (58.9)	144 (50.0)	
GA	92 (37.1)	118 (40.9)	
AA	10 (4.0)	26 (9.0)	0.021
rs1045176 *G/T*			
Allele			
T	402 (81.0)	452 (78.4)	
G	94 (18.9)	124 (21.5)	0.297
Genotype			
TT	161 (64.9)	181 (62.8)	
TG	80 (32.3)	90 (31.2)	
GG	7 (2.8)	17 (5.9)	0.085
rs1045168 *C/T*			
Allele			
T	426 (85.8)	452 (78.4)	
C	70 (14.1)	124 (21.5)	0.001
Genotype			
TT	183 (73.8)	177 (61.4)	
TC	60 (24.2)	98 (34.0)	
CC	5 (2.0)	13 (4.5)	0.002
rs2112703 *A/C*			
Allele			
C	472 (95.1)	524 (90.9)	
A	24 (4.8)	52 (9.0)	0.008
Genotype			
CC	224 (90.3)	238 (82.6)	
CA	24 (9.7)	48 (16.6)	
AA	0 (0.0)	2 (0.7)	0.010
rs2287727 *A/C*			
Allele			
C	329 (66.3)	369 (64.0)	0.473
A	167 (33.6)	207 (35.9)	
Genotype			
CC	114 (45.9)	119 (41.3)	
CA	101 (40.7)	131 (45.4)	0.969
AA	33 (13.3)	38 (13.1)	

Data are shown as n and frequency. *chi-square test. NS: No significant

Supplementary Table S3. In Silico Functional Prediction for Variants DOCK2

**Variant**	**pos (hg38)**	**SiPhy**	**Motifs changed**	**eQTLTarget Gene**	**Tissue**	***p***
rs9307	170083019	Negative	*SETDB1*	*FOXI1*	Testis	8.6×10^{-16}
rs1045168	170077739	Positive	-	*LINC01187*	Lung	8.4×10^{-5}
rs2112703	169700093	Positive	*CTCF,NRSF,Pax-5*	-	-	-

Supplementary Table S4. Allele frequencies (af) of the *DOCK2* SNPs in different populations.

**SNP/Alleles**	**Mexican** (n=288)		**Caucasian*** (n=503)		**Asian*** (n=504)		**African*** (n=661)		**References**
rs2112703 A/C	n	Af (%)	n	Af (%)	n	Af (%)	n	Af (%)	
C	524	90.9	800	79.5	885	87.8	1274	96.4	Present study[A]
A	52	9.0[A]	206	20.5	123	12.2	48	3.6	NCBI*
rs2287727 A/C									
C	369	64.0	280	27.8	394	39.1	34	2.6	Present study[B]
A	207	35.9[B]	726	72.2	614	60.9	1288	97.4	NCBI*
rs1045168 C/T									
T	452	78.4	670	66.6	900	89.3	822	62.2	Present study[F]
C	124	21.5[C]	336	33.4	108	10.7	500	37.8	NCBI*
rs1045176 G/T									
T	452	78.4	874	86.9	547	54.3	521	39.4	Present study[F]
G	124	21.5[D]	132	13.1	461	45.7	801	60.6	NCBI*
rs9307 A/G									
G	406	70.4	656	65.2	712	70.6	1240	93.8	Present study[G]
A	170	29.5[E]	350	34.8	296	29.4	82	6.2	NCBI*

SNP, Single nucleotide polymorphism; Af, Allele frequency (%); *NCBI, National Center for Biotechnology Information (https://www.ensembl.org/index.html (accessed on 20 January 2025)).

[A] The distribution of the rs2112703 *A*, was low in Mexican mestizos, Asian, and African compared to Caucasian population.

[B] The distribution of the rs2287727 *A* allele in Mexican mestizos, was low compared to Caucasian, Asian and African populations.

[C] The distribution of the of the rs1045168 *C* allele in Mexican mestizos and Asian populations was low compared to African and Caucasian populations.

[D] The distribution of the rs1045176 *G* allele in Mexican Mestizos and Caucasian was low when compared to Asian and African populations.

[E] The distribution of the rs9307 *A* allele in Mexican mestizos and Asian, was similar compared to Caucasian and African population.

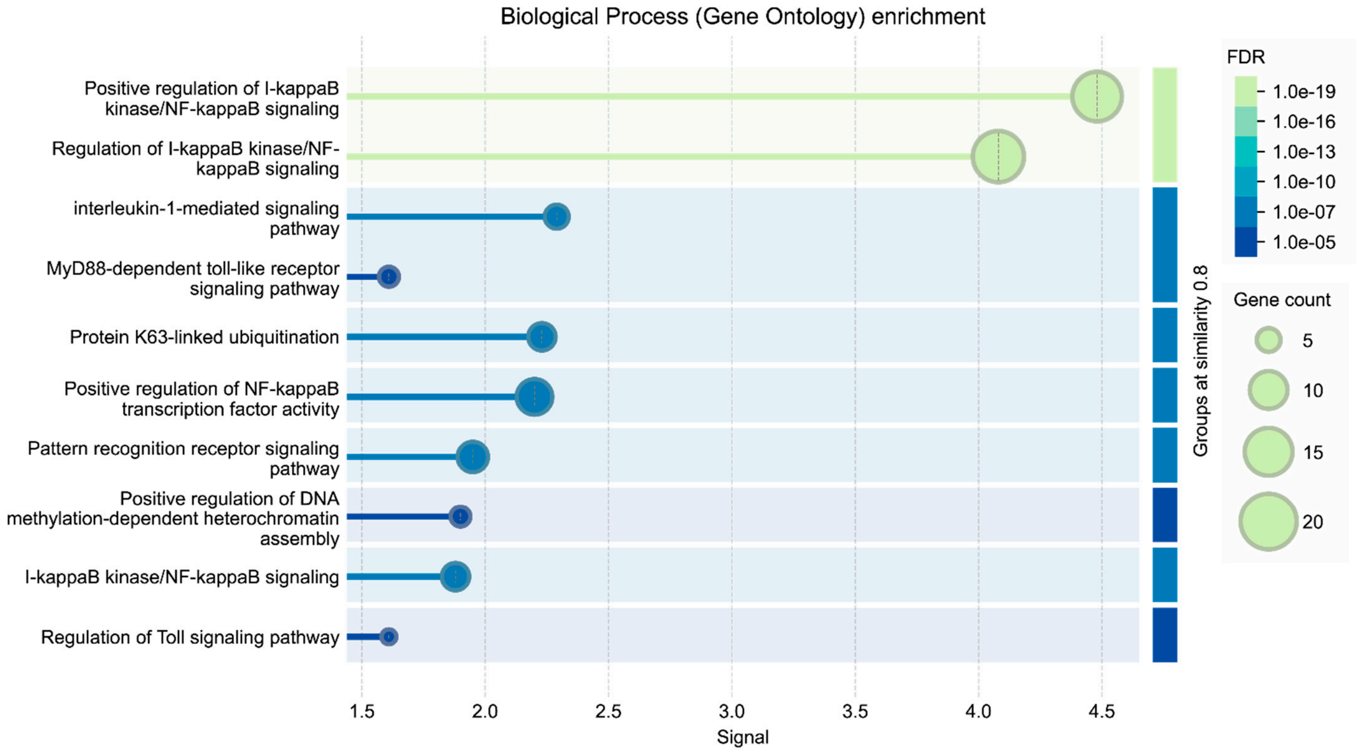

Supplementary Figure S1. *Biological Process enrichment.* The figure represents the functional enrichment analysis of genes within the PPI network, focusing on biological processes (BP). The color gradient indicates the False Discovery Rate (FDR), where lighter green represents highly significant pathways and darker blue denotes less significant ones. Bubble size corresponds to the number of genes associated with each enriched pathway. The analysis highlights key pathways related to immune activation, inflammation, and transcriptional regulation.
